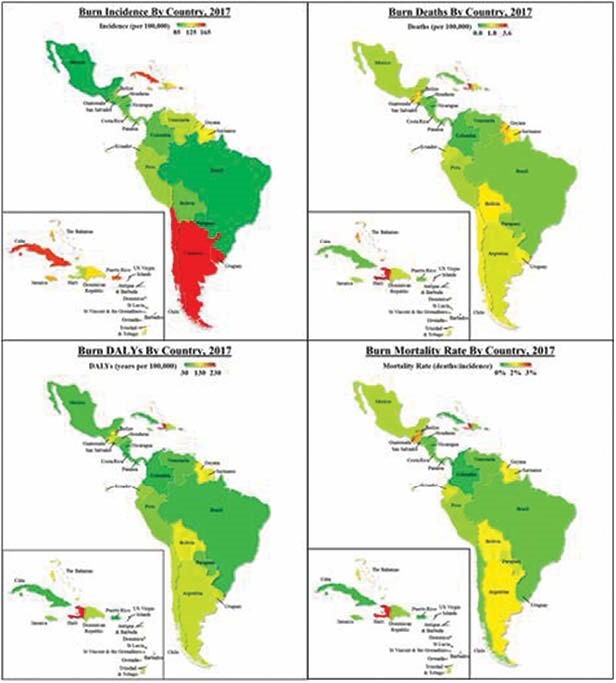# 57 Understanding the Burden of Burn Injury in Latin America & the Caribbean

**DOI:** 10.1093/jbcr/irac012.060

**Published:** 2022-03-23

**Authors:** Zachary J Collier, Alexandre J Bourcier, Priyanka Naidu, William P Magee, Tom Potokar, Justin Gillenwater

**Affiliations:** Keck School of Medicine, University of Southern California, Los Angeles, California; University of California - Los Angeles, Los Angeles, California; University of Cape Town, South Africa, Cape Town, Western Cape; Children’s Hospital Los Angeles, Los Angeles, California; Interburns, Geneva, Geneve; USC/LAC+USC Medical Center, Los Angeles, California

## Abstract

**Introduction:**

Burn injuries are a global health problem disproportionately affecting low- and middle-income countries, especially in Latin America and Caribbean (LAC) where cooking methods, dangerous work conditions, and minimal housing regulations place people at increased risk of burn injury. Until recently, there was limited global epidemiological data on burn injuries. Following publication of the 2017 Global Burden of Disease (GBD17) database, we obtained objective and comparable data on burn injuries while specifically focusing on LAC countries.

**Methods:**

Data from all 35 LAC countries were collected from GBD17 for fire, heat, and hot substance-related injuries to calculate burn incidence, deaths, and Disability-Adjusted Life Years (DALYs) with respect to country, age, and gender from 1990 to 2017. Incidence and deaths were reported as rates per 100,000 persons. Mortality rate was reported as a percentage of deaths-to-incidence. DALYs were reported in years per 100,000 persons. Incidence, death, DALY, and mortality rate trends were assessed using age-standardized, age-stratified, and gender-specific cohorts. All statistical analyses were performed using Excel.

**Results:**

Over 27 years, LAC’s rates for burn incidence (-19%), deaths (-63%), DALYs (-62%), and mortality (-54%) all decreased with the greatest improvements seen in Brazil and Paraguay. All indicators improved around 15% more than the global averages during the same period with LAC’s rates 30-40% below global rates by 2017. The highest burn incidence (227 cases/100,000) was in Southern Latin America (Argentina, Chile, Uruguay). The Caribbean had the worst DALYs (124 years/100,000). In 2017, LAC accounted for 7% of global burns, 5.5% of deaths, and 5.1% of DALYs with Central America contributing the greatest numbers. For 27 years, Chile had the highest burn incidence but Haiti had the greatest death, DALY, and mortality rates of all LAC. Children under 14 years of age and males were disproportionately affected compared to other regional and global cohorts.

**Conclusions:**

Despite a relatively greater reduction in burn severity and lifelong disability within LAC compared to the world, certain regions and countries exhibited significantly higher rates of burn injury, morbidity, and mortality. Central America (e.g. Costa Rica, Belize, Mexico) and the Caribbean (e.g. Haiti, Cuba) were particularly affected, comprising the majority of cases, deaths, and DALYs. This study provides essential analyses for developing regional and country-specific strategies to reduce the burden of burns through targeted interventions for prevention, workforce, and capacity building efforts.